# Job Leaving Intentions of Dentists Associated With COVID-19 Risk, Impact of Pandemic Management, and Personal Coping Resources

**DOI:** 10.3389/ijph.2022.1604466

**Published:** 2022-08-11

**Authors:** Veronika Pacutova, Andrea Madarasova Geckova, Sara Maria Majernikova, Peter Kizek, Andrea F. de Winter, Sijmen A. Reijneveld

**Affiliations:** ^1^ Department of Health Psychology and Research Methodology, Faculty of Medicine, P. J. Safarik University, Kosice, Slovakia; ^2^ Graduate School Kosice Institute for Society and Health, Faculty of Medicine, P. J. Safarik University, Kosice, Slovakia; ^3^ Olomouc University Social Health Institute, Palacky University, Olomouc, Czechia; ^4^ Department of Community & Occupational Medicine, University Medical Center Groningen, University of Groningen, Groningen, Netherlands; ^5^ Division of Biosciences, Faculty of Life Sciences, University College London, London, United Kingdom; ^6^ I. Stomatology Clinic, University Hospital of Louis Pasteur, Faculty of Medicine, P. J. Safarik University, Kosice, Slovakia

**Keywords:** health care workers, COVID-19, quality of life, job leaving intention, pandemic management, personal coping resources, dentists

## Abstract

**Objectives:** The COVID-19 pandemic caused risks and burdens for health professionals and might result in job leaving intentions. To assess the potential risks, we explored the association of the job leaving intentions with exposure to COVID-19 risk, impact of pandemic management on professional and personal life, and personal coping resources among Slovak dentists in the first wave of the outbreak.

**Methods:** We obtained data from 500 dentists (66.8% females, M/SD = 43.8) registered with the Slovak Chamber of Dentists using an online questionnaire. Data were analysed using logistic regression models adjusted for age and gender.

**Results:** Nearly 40% of dentists reported job leaving intentions after the first wave of the pandemic outbreak. Job leaving intentions were associated with exposure to COVID-19 risks (odds ratios, ORs, varying from 1.6 to 4.7), impact of pandemic management on professional and personal life (Ors from 1.6 to 2.9), and personal coping resources (Ors from 0.4 to 0.6).

**Conclusion:** Better management of exposures to risks and impact on professional and personal life, as well as building better personal coping resources may prevent the loss of a valuable workforce in dental care.

## Introduction

Exposure to COVID-19 risk and pandemic-related restrictions seriously increased the burden on health care workers (HCWs) during the first wave, where the total number of cases in Slovakia was 63.080 (until 31 October 2020). This may result in a worsening of their professional and personal quality of life and may even lead to job leaving intentions.

The job leaving intentions of dentists may be reinforced by the burden caused by exposure to the SARS-CoV-2 virus (further: coronavirus) and the particular risks they face in terms of older age and comorbidity [1], as well as the burden due to stigmatization and to information overload [2], all of which affect their well-being. Around 20% of Slovak dentists are aged 65+ years and thus have increased risks, potentially reinforced by a poor health condition [5]. To provide dental care they have to work directly in the oral cavity [3], leading to almost 85% of dentists feeling worried about being infected during their clinical activity [4]. This negative impact of the pandemic was even increased because people around them were afraid as well and considered them to be at increased risk of becoming infected by coronavirus, causing stigmatisation of their profession [6]. Around 30% of people would avoid HCWs simply out of fear of being infected by the coronavirus [7]. COVID-19-related stigma may be facilitated by excessive circulation of information and misinformation [8]. Dentists who followed the pandemic news and were greatly concerned about it [2], and those who were concerned about being infected/infecting others [9–11] were the most affected and burdened [12].

In addition, the negative impact of pandemic management on dentists’ professional and personal quality of life may contribute to their job leaving intentions. Occupational hazards included the burden related to the use of PPE or a lack of PPE, perceived impediments to doing their jobs, insufficient organisational support, and a lack of confidence in protective measures [13]. On the other hand, when PPE was available, bad fitting equipment sometimes led to overheating. Another problematic issue occurred even in the pre-COVID-19 period: a lack of dentists [14] and dental nurses [15]. The shortage of HCWs has increased due to COVID-19 infections among them during the pandemic [16]. The high occupational risk increased work-related stress, which might affect their personal lives too [17]. Perceived levels of stress in HCWs were negatively associated with changes in family relationships, although family support alone was able to mitigate the psychological distress experienced [18]. Further, the main concerns of dentists were economic losses due to the reduced number of elective patients, the financial consequences following the reduction of revenue and the increased costs of acquiring PPE and management of environmental disinfection [3]. More than 70% of dentists reported a worsening of their financial situation and mental well-being [2]. Other contributing factors to HCWs’ poorer quality of life were being parent of dependent children, having an infected family member, experiencing a longer quarantine, and a lack of practical support [19].

Contrary to these negative effects, personal resources may positively influence the handling of difficult pandemic situations of HCWs and potentially protect them from having job leaving intentions. Altruistic acceptance of work-related risk has been shown to have protective effects on mental well-being [20, 21]. The resilience of HCWs was higher in men than in women and seniority proved to be another protective factor. The ability to cope well with the burden caused by COVID-19 was aided by adequate rest, clear communication, sharing stress, resilience, personal protection, and ethical support for treatment decisions among frontline HCWs [19, 22].

Actual job leaving by dentists will increase the shortage that already exists in many countries. To assess the potential risks of this, we explored the association of the job leaving intentions of dentists with exposure to COVID-19 risk, impact of pandemic management on professional and personal life, and personal coping resources among Slovak dentists in the first wave of the pandemic outbreak.

## Methods

### Sample and Procedures

We invited all Slovak practising dentists (N = 3,884), who also have the legal obligation to be members of the Slovak Chamber of Dentists, to participate in an online survey via the umbrella Chamber in October 2020. The questionnaire we used was developed in cooperation with representatives of the participating dentists. We interviewed them and extracted issues relevant for exposure to COVID-19 risk, the impact of pandemic management on their professional and personal life, and the personal coping resources they had available. Based on that, we prepared appropriate measurements, adapted their comments and piloted the final version to assure clarity and suitability. The online invitation to participate in a survey via the Slovak Chamber of Dentists resulted in a response rate of 12.9%, in which the age and gender composition of our sample stayed quite similar to the target population, e.g., dentists registered with the Slovak Chamber of Dentists. Our sample had a slightly higher percentage of females than in the target population (66.8% vs. 61.4%), and fewer dentists over 66 years old (10.0% vs. 20.1%), the latter group being very small [2]. From 635 responses we excluded those who did not fill in any questions (*n* = 15), those who did not report their gender (*n* = 113), and those who had a different profession than dentists (2 nurses, 5 others). The final sample included 500 respondents (66.8% females, mean age/SD = 43.8/14.4). A post hoc analysis indicated that with our final sample size we could detect the explored associations with job leaving intention with a power of 95.5% at *p* = 0.05.

### Measures

Respondents were asked if they had interrupted the provision of dental care or their clinic, or if they were considering closing their clinic at the time, with the answer’ options: 1) I quit my job; 2) I am considering quitting my job; 3) I interrupted dental care provision; 4) I am considering interrupting dental care provision; 5) I reduced dental care provision; 6) I am considering reducing dental care provision and 7) I am not considering reducing dental care provision. Those responding that they had quit/interrupted or reduced the provision of dental care or were considering quitting, interrupting, or reducing dental care provision were assigned as having job leaving intentions*.*

Exposure to COVID-19 risk was measured by asking respondents about (1) being at risk of COVID-19 (being in a risk group due to being 65+ years old or having a poor health condition vs. being without risk), (2) exposure to coronavirus (being infected or knowing anybody who was infected vs. not being exposed to infection), (3) exposure to quarantine (the respondents themselves or close relatives being quarantined vs. not being quarantined), (4) risk perceptions (the mean score of 7 items covering risk perception varied between 1 and 5; a higher score represents higher risk perception [23, 24]), (5) being stigmatised (people avoid the respondents and their family due to their work during the pandemic vs. not being stigmatised), (6) information overload (following pandemic news several times per day and being highly concerned, following pandemic news several times per day or being very concerned vs. following pandemic news and not being concerned), (7) availability of PPE and ability to implement anti-pandemic measures (not enough PPE and unable implement measures, not enough PPE or unable implement measures, enough PPE and able to implement measures).

The impact of pandemic management on professional and personal life was assessed by a set of items covering the pandemic-related limitations of providing health care, an item on concern about poor health care provision, and sets of items covering impact on quality of life [2]. Respondents were asked how much they were hindered in providing health care in the original quality during the first lockdown (March-June 2020) due to (a) a lack of PPE, (b) infection risk in the work environment, (c) obligatory safety measures, (d) a lack of staff and (e) client concerns in order to assess pandemic related limitations of provided health care. Answers on a Likert scale were dichotomised as partially/not limited vs. limited/significantly limited for each subcategory to enable a comparison between individuals who perceived a rather limited or no impact of pandemic management versus a stronger impact of pandemic management. The concern about poor health care provision was assessed by asking respondents whether the health care condition of their regular patients would worsen due to pandemic measures related to the possibilities of dental care provision (certainly agree/rather agree vs. rather disagree/certainly disagree). The impact on quality of life was measured by asking respondents if difficulties in providing health care due to introducing pandemic management affected their (a) family life and activities, (b) housekeeping, (c), relationships with close relatives, (d) financial situation and (e) mental well-being during the first wave of the COVID-19 pandemic. Answers on a Likert scale were dichotomized into those reporting worsening (slightly/significantly worsened) and improving (slightly/significantly improved vs. did not change) effects in a particular area of life.

We measured personal coping resources as optimism (certainly agree or agree that we would successfully handle the pandemic vs. disagree or certainly disagree), sacrifice (certainly agree or agree to provide dental care during the COVID-19 pandemic just to avoid to worsening of their patients’ health care condition vs. disagree or certainly disagree), altruistic acceptance of risk associated to COVID-19 (certainly agree or agree that they accepted the risk associated with dental care provision to COVID-19 patients vs. disagree or certainly disagree), and resilience (the mean score varies between 1 and 5; a higher score represents higher resilience The Brief Resilience Scale [25]) (Supplementary Appendix SA).

### Statistical Analysis

First, we described the exposure to COVID-19 risk, impact of the pandemic on professional and personal life, personal coping resources, and job leaving intentions of Slovak dentist as rates, means, and standard deviations (SDs). Second, we assessed the association of job leaving intentions with exposure to COVID-19 risk, impact of pandemic management on professional and personal life, and personal coping resources using logistic regression models adjusted for age (continuous level variable, centred age and age squared) [48] and gender per each variable separately. We used IBM SPSS Statistics 23 for Windows for all analyses.

## Results

### Background Characteristics

Most dentists in our sample were owners of private dental clinics 77.8% (*n* = 389), while 28.2% (*n* = 141) work at private clinics and only 2.0% (*n* = 10) were from state dental clinics. Similarly, the majority of them were traditional dentists 86.2% (*n* = 431), who treated adult and child clients 93.4%, (*n* = 467). For more details, see Table 1.

**TABLE 1:** Demographic characteristics of the respondents (Slovakia 2020; *n* = 500 dentists).

Variables	N (%)
Age (mean/SD)	43.8/14.4
Gender	
Women	334 (66.8)
Men	166 (33.2)
Type of dental clinic	
Private dental clinics—owners	389 (77.8)
Private dental clinics—employees	141 (28.2)
State dental clinics	10 (2.0)
Specialization of dentists	
Traditional dentists	431 (86.2)
Specialized dentists	58 (11.6)
Other	11 (2.2)
Type of patients treated	
Adults and children	467 (93.4)
Only adults	24 (4.8)
Only children	9 (1.8)

Note: Some dentists were owners and employees at the same time.

### Exposure to COVID-19 Risk, Impact of the Pandemic, Personal Coping Resources, and Job Leaving Intentions

A considerable portion of dentists experienced risks due to COVID-19, with rates being highest for stigmatisation (62.6%) and being exposed to COVID-19 (48.8%). Regarding the impact on their professional and personal life, dentists experienced the most concerns about poor health care provision (68.8%) and the worsening of their financial situation (85.4%). Quite a lot of them were optimistic (84.1%) and altruistic (71.8%) about the pandemic. Job leaving intentions were reported by 39.6% of the dentists. For more details, see Table 2.

**TABLE 2:** Exposure to COVID-19 risk, impact of the pandemic on professional and personal life, and personal coping resources of Slovak dentists (Slovakia 2020; *n* = 500 dentists).

Variables	N (%)
Exposure to COVID-19 risk	
Being at risk of COVID-19 due to being aged 65 + or a poor health condition	148 (29.6)
Being exposed to coronavirus	241 (48.8)
Being exposed to quarantine	140 (28.3)
Risk perception	3.60 (0.03)^a^
Being stigmatised	219 (62.6)
Information overload	
Followed the news several times per day or being very concerned	146 (30.7)
Followed the news several times per day and being very concerned	93 (19.6)
Availability of PPE and ability to implement anti-pandemic measures	
Not enough PPE or unable to implement measures	128 (28.1)
Not enough PPE and unable to implement measures	222 (48.7)
Impact on professional life	
Providing health care limited due to lack of PPE	200 (54.9)
Providing health care limited due to infection risks in the work environment	219 (62.6)
Providing health care limited due to obligatory safety measures	169 (49.6)
Providing health care limited due to lack of staff	131 (38.8)
Providing health care limited due to client concerns	164 (47.1)
Concern about poor health care provision	267 (68.8)
Impact on personal life	
Worsening of family life and activities	190 (45.0)
Worsening of housekeeping	149 (35.4)
Worsening of relationships with relatives	89 (21.1)
Worsening of financial situation	362 (85.4)
Worsening of mental well-being	298 (70.3)
Personal coping resources	
Being optimistic about pandemic handling	306 (84.1)
Willingness to provide care to prevent a worsening of patients’ health condition	297 (59.4)
Altruistic acceptance of risk associated with COVID-19 patients	326 (71.8)
Resilience	3.27 (0.04)^a^
Having Job Leaving Intention	170 (39.6)

a Mean (SD).

### Associations of Job Leaving Intentions With Exposure to COVID-19 Risk, Impact of Pandemic Management on Professional and Personal Life, and Personal Coping Resources

Job leaving intentions were more likely to be reported by dentists who belonged to a risk group because of their age and comorbidity (odds ratio/95% confidence interval, OR/CI: 3.2/1.97–5.31), or because they perceived a higher risk of COVID-19 (4.7/3.15–7.06) than dentists who did not report any risk. Moreover, we found job leaving intentions to be increased due to a large number of factors, as shown by odds ratios in a range of 1.6–2.9. This regarded dentists who felt stigmatisation or were concerned about information overload and health care quality or the high risk work environment. It also included those who experienced the unavailability of PPE and the inability to implement measures or a worsening of their personal life (in housekeeping, relationships, financial situation, and mental well-being). Less strong associations with job leaving intentions were seen in other areas, such as their exposure to COVID-19 risk, a lack of staff, or a worsening of family life and activities (1.6/1.05–2.34; 1.9/1.19–3.00; 1.6/1.10–2.45). Regarding personal coping strategies, job leaving intentions were less likely for dentists with higher resilience (0.6/0.42–0.77), more altruism (0.6/0.37–0.89), sacrifice (0.5/0.31–0.81) and optimism (0.4/0.24–0.76). In addition, the age of our dentists was significant for almost every researched factor, except for those who belonged to a risk group, in contrast with gender, which was statistically non-significant. For more details, see Table 3.

**TABLE 3:** Association of job leaving intentions with exposure to COVID-19 risk and with the impact of pandemic management on professional and personal life (Slovakia 2020; *n* = 500 dentists), crude (model 1) and adjusted for age and gender (model 2).

Variables	Job leaving intention OR (95%CI)	
	Model 1 (crude)	Model 2 (adjusted)
Exposure to COVID-19 risk		
Being at risk of COVID-19	**3.4 (2.24–5.28)**	**3.2 (1.97–5.31)**
Being exposed to coronavirus	1.4 (0.93–2.03)	**1.6 (1.05–2.34)**
Being exposed to quarantine	1.2 (0.81–1.89)	1.3 (0.82–1.94)
Risk perception	**4.8(3.23–7.20)**	**4.7 (3.15–7.06)**
Stigmatisation	**2.6(1.55–4.32)**	**2.9(1.69–4.87)**
Information overload		
Followed the news or highly concerned	1.5 (0.95–2.34)	1.5 (0.94–2.34)
Followed the news and highly concerned	**2.3 (1.35–3.77)**	**2.2 (1.30–3.68)**
Availability of PPE and ability to implement anti-pandemic measures		
Not enough PPE or unable to implement measures	**2.2 (1.26–3.92)**	**2.3 (1.31–4.15)**
Not enough PPE and unable to implement measures	**1.7 (1.02–2.89)**	**1.8 (1.08–3.11)**
Impact on professional life		
Lack of PPE	**2.0 (1.27–3.03)**	**2.0 (1.26–3.08)**
Infection risk in the work environment	**2.8 (1.73–4.48)**	**2.9 (1.77–4.72)**
Obligatory safety measures	1.3 (0.81–1.94)	1.2 (0.76–1.87)
Lack of staff	**1.8 (1.12–2.74)**	**1.9 (1.19–3.00)**
Client concerns	1.4 (0.88–2.09)	1.4 (0.92–2.23)
Concern about poor health care provision	**2.3 (1.46–3.74)**	**2.6 (1.60–4.21)**
Impact on personal life		
Worsening of family life and activities	**1.7 (1.13–2.49)**	**1.6 (1.10–2.45)**
Worsening of housekeeping	**2.1 (1.39–3.15)**	**2.1 (1.41–3.24)**
Worsening of relationships with close relatives	**2.2 (1.39–3.60)**	**2.2 (1.36–3.57)**
Worsening of financial situation	**2.7 (1.42–5.18)**	**2.6 (1.32–4.90)**
Worsening of mental well-being	**2.2 (1.40–3.51)**	**2.2 (1.41–3.58)**
Personal coping resources		
Being optimistic about pandemic handling	**0.5 (0.25–0.80)**	**0.4 (0.24–0.76)**
Willingness to provide care	**0.5 (0.30–0.79)**	**0.5 (0.31–0.81)**
Acceptance of risk associated with COVID-19 patients	**0.6 (0.39–0.92)**	**0.6 (0.37–0.89)**
Resilience	**0.6 (0.44–0.79)**	**0.6 (0.42–0.77)**

Significant values are in bold.

## Discussion

We explored the associations of job leaving intentions with exposure to COVID-19 risk, impact of pandemic management on professional and personal life, and personal coping resources in Slovak dentists during the first wave of the COVID-19 pandemic. We found that nearly 40% of dentists reported job leaving intentions after the first wave of the pandemic outbreak and also confirmed that the explored areas were associated as increasing job leaving intentions among Slovak dentists. Moreover, we found that personal coping resources were able to decrease the chance of their job leaving intentions.

We found job leaving intentions in particular to be related to risks due to older age (65+) or a poor health condition, perceived risk, being stigmatised, being overloaded by information and not being able to protect themselves sufficiently. These findings are in line with those of Sibbald et al., showing that the age of doctors was the main factor associated with increased job leaving intentions [26, 27]. Generally, during the COVID-19 pandemic employees in Slovakia showed intentions to leave their job 4x more often than before the pandemic [28]. In our sample, 20% of Slovak dentists are 65 + years old and this may increase health risks during the COVID-19 pandemic; thus, it is very likely that these older dentists considered retiring. In line with this, Portoghese et al. found that higher age, perception of higher COVID-19 risk, and the stigmatisation of HCWs had an impact on perceived job stress, which might lead to the retirement intentions of dentists [29, 30]. Dentists who were older, with a poorer health condition, perceiving higher risk or stigmatisation without the possibility to protect themselves and following the pandemic news, which concerned them, might be more likely to consider leaving the profession.

We found job leaving intentions to be further associated with the impact of COVID-19 on professional life due to a lack of PPE, infection risk in the work environment, a lack of staff, and concern about the worsening of patients’ health care condition during the pandemic. The findings are consistent with the study by Cole et al., who found that a shortage of PPE increased the job leaving intentions of Alabama nurses [31]. A study by Vick and Muhic et al. showed that dentists missing dental support staff were less satisfied and more likely to leave their profession, which might also reflect the lack of staffing during the pandemic [32, 33]. Moreover, another study by Sasso et al. pointed out that a shortage of HCW staff could be one of the push factors in job leaving intentions due to the increased workload [34]. The increased workload of dentists could also be caused by sickness leave or other employee outages of their nurses, the replacement of whom is usually very problematic [15], and quick replacement during the pandemic period could be almost impossible. Dentists who felt limitations in health care provision due to a lack of PPE or staff, concerning poor health care provision, and a high-risk work environment might be more likely to consider leaving their profession.

We found job leaving intentions to be associated with a worsening of personal life due to all the explored dimensions (from family life to mental well-being). This is in line with findings of Degen et al., who pointed out that work-family conflicts particularly increased intentions to leave a job for women [35], who also made up a majority in our sample. Women carry a greater burden and risk in the application of anti-epidemic measures [36, 37], due to the closure of kindergartens and schools as well as supply problems, but also the restriction of movement itself brings a greater risk of stress and exhaustion. The impact on their personal life might increase the job dissatisfaction of dentists, which might accelerate their thoughts about leaving the profession [33]. Another study by Hopcraft et al. pointed out the disproportionate number of female dentists who receive a lower salary than male dentists, regardless of experience, which was the main reason for their considering leaving [38]. The longer restrictive measures are extended, the more likely they are to affect the economic capacity of dental practices, which may translate into job losses and qualitative and quantitative changes in care provision [39]. Many dental clinics temporarily closed or only permitted limited emergency services, leading to serious financial problems for dentists [10], which might limit their ability to restore dental services after the curbing of the pandemic surge. In line with this and our findings, the financial situation was associated with job insecurity and increased psychological distress in Italian dentists in the study by Gasparro et al. [40]. Psychological distress associated with the pandemic outbreak and its management was confirmed in many other studies [41, 42]; thus, our findings are in line with this. Dentists whose personal lives are worsening due to the COVID-19 pandemic might be more likely to consider leaving the profession.

Personal resources, such as being optimistic about the pandemic handling, a willingness to provide care to prevent the worsening of patients’ health conditions, altruistic acceptance of COVID-19-related risk, and higher resilience, were associated with a lower chance of reporting job leaving intentions. Altruistic acceptance of job-related risk protected HCWs from negative psychological outcomes following the outbreak of SARS [24, 43] and decreased the job leaving intentions of dentists [Bolin]. Our findings suggest that further characteristics may prevent job leaving intentions. This aligns with other findings that higher resilience prevents job leaving intentions via enhanced work engagement and less loss of compassion fatigue in other studies [44, 45]. This could be associated with the fact that over the years of their professional experience, dentists built defence mechanisms to protect themselves. They thus had more freedom and were more relaxed, thanks to the lessons learned over years of practice [46]. Activation of personal coping resources to cope with the burden related to the critical period of the pandemic may protect HCWs and prevent a loss of workforce.

### Strengths and Limitations

The main strength of this study is that we reached a large national sample of dentists during the first wave of the COVID-19 pandemic. Based on this, we were able to gather information about job leaving intentions, exposure to COVID-19 risk, impact on professional and personal life, and personal coping resources. On the other hand, some limitations should also be considered. Our study was cross-sectional, so no causal relations could be established between job leaving intentions and risk perception.

### Implications

A broad range of factors related to exposure to COVID-19 risk, impact on professional and personal life, and personal coping resources contributed highly to job leaving intentions among Slovak dentists. This implies that better management of such hazards might decrease exposure to risk and prevent undesirable impacts on job leaving intentions. This includes not only minimalizing the exposure to coronavirus or the protection of dentists, but also decreasing the negative impact of pandemic management on their professional and personal lives by means such as adequate access to PPE, sufficient staffing, providing a more comprehensive management strategy, including the training of HCWs (proven in the study by Labrague and De los Santos [47]), and using a communication strategy that prevents information overload and stigmatisation. Economic losses resulting from restrictive measures need to be compensated to overcome the troubles in maintaining the provision of dental care. As personal coping resources help to battle mental health challenges in difficult times like a pandemic outbreak, their development should be a part of the professional training of HCWs. The potential high impact on job leaving in this already poorly staffed sector shows a strong need for further action.
