# Supplementary material for: Job Leaving Intentions of Dentists Associated With COVID-19 Risk, Impact of Pandemic Management, and Personal Coping Resources
Source: Int J Public Health. 2022 Aug 11;67:1604466. doi: 10.3389/ijph.2022.1604466 (PMC9413052; doi:10.3389/ijph.2022.1604466)
Supplement: Supplementary file 1 [file DataSheet1.docx]

# Supplementary Material – Questionnaire

**Job Leaving Intention**

Q.1 Did you consider or quit/interrupt or reduce dental care provision?

- I quit dental care provision
- I am considering quitting dental care provision
- I interrupted dental care provision
- I am considering interrupting dental care provision
- I reduced dental care provision
- I am considering reducing dental care provision
- No, I am not considering any changes in dental care provision

**Being at COVID-19 risk**

Q.1 Do you belong to a COVID-19 risk group?

- Yes, I am over 65+
- Yes, I have a poor health condition (chronic disease, immunocompromising disease)
- No

**Exposure to COVID-19**

Q.1 Have you or any who you know (close relatives, friends) been diagnosed with COVID-19?

- I do not know anybody, who has been diagnosed with COVID-19.
- I know somebody, who has been diagnosed with COVID-19.
- I have been diagnosed with COVID-19.

**Exposure to quarantine**

Q.1 Have you or any of your close relatives been quarantined due to possible contact with a person infected with COVID-19?

- Yes
- No

**Risk Perceptions** (7 items)

Q.1 I believed that my job was putting me at great risk.

- Certainly agree
- Agree
- Not sure
- Disagree
- Certainly disagree

Q.2 I felt extra stress at the work.

- Certainly agree
- Agree
- Not sure
- Disagree
- Certainly disagree

Q.3 I was afraid of falling ill with COVID-19.

- Certainly agree
- Agree
- Not sure
- Disagree
- Certainly disagree

Q.4 I felt I had little control over whether I would get infected or not.

- Certainly agree
- Agree
- Not sure
- Disagree
- Certainly disagree

Q.5 I thought I would be unlikely to survive if I were to get COVID-19.

- Certainly agree
- Agree
- Not sure
- Disagree
- Certainly disagree

Q.6 I was afraid I would pass COVID-19 on to others.

- Certainly agree
- Agree
- Not sure
- Disagree
- Certainly disagree

Q.7 My family and friends were worried that they might get infected through me.

- Certainly agree
- Agree
- Not sure
- Disagree
- Certainly disagree

**Stigmatisation:**

Q.4 Society avoids my family because of origin of the dentistry profession during pandemic:

- (a, certainly agree; b, agree; c, not sure; d, disagree; e, certainly disagree)

**Information overload:**

Q.1 Did you follow the pandemic news during the first lockdown?

• Yes, several times per day

• Yes, at least once a day

• Yes, but not more than usual

• No

Q.2 Were you concerned about the pandemic news?

• No

• A little concerned

• Very concerned

**Availability of PPE and ability to implement anti-pandemic measures:**

Q.1 During the first lockdown, did you have at your disposal all the personal protective equipment needed?

• Personal contact with clients was excluded

• In personal contact with clients, we were able to fully assure COVID protection

• In most contacts with clients, we were unable to ensure sufficient protection

• During some periods, we had to work with clients in insufficiently protected conditions

Q.2 How much were you able to implement and maintain COVID-related recommendations during the first lockdown?

• We were unable to implement them fully

• We were able to implement them fully

• We were unable to implement them at all

**Impact on professional life:**

Q.1 How much did the following circumstances hinder you in providing health care in the original quality?

(a, limited; b, significantly limited; c, partially limited; d, not limited)

• Lack of PPE

• Infection-risks in the work environment

• Obligatory safety measures

• Lack of staff

• Client concerns

**Concern about poor health care provision:**

Q.2 I think that pandemic measures could potentially cause a worsening of patients’ health care condition.

(a, certainly agree; b, rather agree; c, rather disagree; d, certainly disagree)

**Impact on personal life:**

Q.1 Did difficulties in providing health care due to introducing pandemic management affect:

(a, significantly improved; b, slightly improved; c, did not change; d, significantly worsen)

• Your family life and activities

• Your housekeeping

• Relationships with relatives

• Your financial situation

• Your mental well-being

**Optimism:**

Q.1 Do you think we will successfully handle the pandemic in Slovakia?

(a, certainly yes; b, yes; c, no; d, certainly no)

**Sacrifice:**

Q.2 I decided to provide dental care even during the pandemic measures simply to avoid the worsening of patients’ health care condition.

(a, certainly agree; b, rather agree; c, rather disagree; d, certainly disagree)

**Altruism:**

Q.3 I accept the risk associated with dental care provision to COVID-19 patients:

(a, certainly agree; b, agree; c, not sure; d, disagree; e, certainly disagree)

**Resilience:** (3 items)

Q.1 I have a hard time making it through stressful events.

(a, strongly disagree; b, disagree; c, neutral; d, agree; e, strongly agree)

Q.2 It is hard time for me to snap back when something bad happens.

(a, strongly disagree; b, disagree; c, neutral; d, agree; e, strongly agree)

Q.3 I tend to take a long time to get over setbacks in my life.

(a, strongly disagree; b, disagree; c, neutral; d, agree; e, strongly agree)
